# High methylation levels of *PCDH10* predict poor prognosis in patients with pancreatic ductal adenocarcinoma

**DOI:** 10.1186/s12885-019-5616-2

**Published:** 2019-05-14

**Authors:** Maria Cristina Curia, Fabiana Fantini, Rossano Lattanzio, Francesca Tavano, Francesco Di Mola, Mauro Piantelli, Pasquale Battista, Pierluigi Di Sebastiano, Alessandro Cama

**Affiliations:** 1 Department of Medical, Oral and Biotechnological Sciences,‘G. d’Annunzio’ University, Via dei Vestini n.31, 66100 Chieti, Italy; 2 Center of Excellence on Aging and Translational Medicine (CeSi-Met), “G. d’Annunzio” University, Chieti, Italy; 3 Division of Gastroenterology and Research Laboratory, IRCCS “Casa Sollievo della Sofferenza”, San Giovanni Rotondo, Italy; 4 Division of Surgical Oncology “SS Annunziata” Hospital, Chieti, Italy; 5 Department of Pharmacy,‘G, d’Annunzio‘ University, Chieti, Italy

**Keywords:** Pancreatic ductal adenocarcinoma, Epigenetics, Protocadherins, *PCDH10*, DNA methylation, Survival, Combined bisulfite restriction analysis (COBRA), mRNA expression

## Abstract

**Background:**

Pancreatic ductal adenocarcinoma (PDAC) is one of the most lethal malignancies and is not a clinically homogeneous disease, but subsets of patients with distinct prognosis and response to therapy can be identified by genome-wide analyses. Mutations in major PDAC driver genes were associated with poor survival. By bioinformatics analysis, we identified protocadherins among the most frequently mutated genes in PDAC suggesting an important role of these genes in the biology of this tumor. Promoter methylation of protocadherins has been suggested as a prognostic marker in different tumors, but in PDAC this epigenetic modification has not been extensively studied. Thus, we evaluated whether promoter methylation of three frequently mutated protocadherins, *PCDHAC2, PCDHGC5* and *PCDH10* could be used as survival predictors in PDAC patients.

**Methods:**

DNA extracted from 23 PDACs and adjacent non-neoplastic pancreatic tissues were bisulfite treated. Combined Bisulfite Restriction Analysis (COBRA) coupled to denaturing high-performance liquid chromatography (dHPLC) detection and bisulfite genomic sequencing (BGS) were used to determine the presence of methylated CpG dinucleotides in the promoter amplicons analyzed.

**Results:**

In an exploratory analysis, two protocadherins showed the same pattern of CpG methylation in PDAC and adjacent non-neoplastic pancreatic tissues: lack of methylation for *PCDHAC2*, complete methylation for *PCDHGC5*. Conversely, the third protocadherin analyzed, *PCDH10*, showed a variable degree of CpG methylation in PDAC and absence of methylation in adjacent non-neoplastic pancreatic tissues. At Kaplan–Meier analysis, high levels of *PCDH10* methylation defined according to the receiver operating characteristic (ROC) curve analysis were significantly associated with worse progression-free survival (PFS) rates (*P* = 0.008), but not with overall survival (OS). High levels of *PCDH10* methylation were a prognostic factor influencing PFS (HR = 4.0: 95% CI, 1.3–12.3; *P* = 0.016), but not the OS.

**Conclusions:**

In this study, we show for the first time that the methylation status of *PCDH10* can predict prognosis in PDAC patients with a significant impact on the outcome in terms of progression-free survival. High levels of *PCDH10* promoter methylation could be useful to identify patients at high risk of disease progression, contributing to a more accurate stratification of PDAC patients for personalized clinical management.

## Background

Pancreatic ductal adenocarcinomas (PDAC) arise from the exocrine pancreas, account for 95% of pancreatic cancers and, due to the poor survival rate, represent the seventh leading cause of cancer-related deaths worldwide and the third in the United States [1]. PDAC are typically diagnosed at advanced stages when the only available treatments are palliative. The poor clinical outcome of PDAC is attributable to early local spread, the high trend of distant metastasis, and resistance to radio- and chemotherapy [2]. A better understanding of molecular and epigenetic events affecting progression and response to therapy has the potential to improve early diagnosis, prognostic evaluations, and to provide new elements for rational therapeutic approaches.

Some studies analyzed the mutational landscape of PDAC using state of the art genomic sequencing [3–6]. Conversely, the characterization of epigenetic changes occurring in PDAC has not been extensively studied. A comprehensive study analyzed genome-wide promoter methylation in pancreatic cell lines with the aim to improve the diagnosis of PDAC and to identify key regulatory genes and pathways that merit therapeutic targeting [7]. A subset of CpG island showing aberrant methylation in cell lines was also investigated in PDAC tumor specimens, but the levels of methylation often differed from that observed in cell lines [7]. Considering the importance of epigenetic changes in malignant transformation, further characterization of these alterations in PDAC tumor specimens is needed.

Genes that are frequently mutated in PDAC are likely to play an essential role in the biology of this tumor, and they might also be a target of epigenetic dysregulation. Therefore, studying epigenetic changes in these genes may provide complementary evidence of their role in PDAC malignant transformation.

Protocadherins were included in the homophilic cell adhesion gene set that was shown to be subject to frequent alterations in an early study on transcriptome sequencing pancreatic cancers [8], but this observation was not highlighted in subsequent genome-wide studies [3–6]. These genes are among those showing aberrant methylation in pancreatic cancer cell lines [7], suggesting their relevant role in PDAC carcinogenesis. Protocadherins represent a major subfamily of the cadherin superfamily [9, 10] and more than seventy coding genes for protocadherins have been identified. Based on their organization, their protein products can be divided into two large groups: “*clustered*” and “*non clustered*” protocadherins [11]. The *clustered* protocadherins constitute the largest group. Unlike the *clustered*, the *non clustered* protocaderins are so named because their genes are not located in a single gene locus, but in three different chromosomal loci. They contain six extracellular cadherin domains, a transmembrane domain and a cytoplasmic tail differing from that of the classical cadherins [10]. Protocadherins exhibit cell-to-cell adhesion activities, but distinct from that of classical cadherins, and are believed to possess other important functions such as signal transduction and growth control, although the exact mechanisms of action have not been fully elucidated. Different studies indicated a potential role as tumor suppressors for some of them [12]. The onset and the malignant progression of different cancers are often associated with the lack of expression of protocadherins caused by an epigenetic silencing event that involves hypermethylation of specific chromosomal regions [13]. Promoter methylation of protocadherins has been suggested as a prognostic marker in different tumors, including prostate, gastric, colorectal, bladder and clear cell renal cell carcinoma [13], but in PDAC this epigenetic modification has not been extensively studied. In particular, only *PCDH10* had been previously studied in PDAC primary tumors, but that study failed to find any correlation between *PCDH10* methylation status and tumor staging [14].

Considering that protocadherins are frequently mutated in PDAC [8] and could play a crucial role in the biology of this tumor, but little is known about their epigenetic modifications, we analyzed promoter methylation of three protocadherins. In particular we analyzed promoter CpG methylation of *PCDH10*, *PCDHAC2* and *PCDHGC5* that in our query of The Cancer Genome Atlas database resulted among the most frequently mutated in PDAC. Notably, *PCDH10* promoter methylation had been previously suggested as a prognostic marker in prostate, gastric and colorectal cancer [13]. In our study, *PCDH10* methylation was identified as a factor associated with PDAC progression-free survival and, consequently, we suggest its possible role as a prognostic marker that might be useful for personalized treatment.

## Methods

### Patients samples

Samples from surgically resected primary PDAC were collected from a series of 23 patients recruited at the Department of Surgery of “Casa Sollievo della Sofferenza” Hospital, IRCCS San Giovanni Rotondo. Only patients with histologically proven primary PDAC were enrolled in the study. Exclusion criteria for patients were a previous diagnosis for PDAC and neoadjuvant treatment before surgery. Tumors were staged in accordance with the TNM classification [15]. Clinical features and tumor characteristics were reported in Table 1. Patients gave informed written consent and approval from the ethical committee of the “Casa Sollievo della Sofferenza” IRCCS, San Giovanni Rotondo was obtained. In DNA methylation analyses Capan-2 human pancreatic cancer cell line was used as a control fully methylated for *PCDH10* [7]. For *PCDH10* mRNA expression analysis we used *PCDH10* fully methylated pancreatic (Capan-2, AsPC-1) and gastric (AGS) cancer cell lines, as well as *PCDH10* unmethylated breast cancer cell line (MB-231) [16, 17].

**Table 1:** Patients and tumor characteristics (*n* = 23)

Variable	Value (%)
Age at diagnosis (yr)	
Median	67.0
Range	38–78
Gender	
Male	11 (47.8)
Female	12 (52.2)
Tumor location	
Head	21 (91.3)
Body	1 (4.3)
Tail	1 (4.3)
Tumor stage	
I	0 (0.0)
II	6 (28.6)
III	15 (71.4)
IV	0 (0.0)
LN metastasis	
No	6 (26.1)
Yes	17 (73.9)
*PCDH10* methylation status	
Low	16 (69.6)
High	7 (30.4)
Tumor progression	
No	8 (34.8)
Yes	15 (65.2)
Occurrence of death	
No	5 (21.7)
Yes	18 (78.3)

## Promoter methylation analysis

### DNA extraction and bisulfite modification of DNA

Resected PDACs and adjacent non-neoplastic tissues from the same patients were taken separately, immediately frozen in liquid nitrogen and stored at − 80 °C until the nucleic acid extraction. These control tissues were verified as tumor-free by a pathologist.

Genomic DNA was isolated using the AllPrep DNA/RNA mini kit (Qiagen, Hilden, Germany) according to the manufacturer’s instructions. DNA concentration and purity were controlled by NanoDrop Spectrophotometer (Thermo Fisher, Waltham, MA, USA).

Bisulfite treatment was performed according to the manufacturer’s protocol (EpiTect Bisulfite Kit, Qiagen). The bisulfite-treated DNA was amplified with primers designed according to MethPrimer [18]. Primer sequences and PCR conditions are available in Table 2.

**Table 2:** Sequences of primers employed for PCR amplification of bisulfite-treated DNA

gene	Amplicon (bp)	CpG n.	Sequence 5′- 3′
*PCDHAC2*	235	11	f.aggggtttgattgttttttttagat r.actcaacaaatcctactctaattc
*PCDHGC5*	290	14	f.gggtatggtgttatttagtttaat r.ccaaactctaaaatcactataatat
*PCDH10*	196	16	f. ggttagggaggatggatgtaagtat r. cccaccatactaaattaaaccactaat

### Combined bisulfite restriction analysis (COBRA)

COBRA is a technique to semiquantitate the methylated and unmethylated DNA after sodium bisulfite treatment by using restriction enzyme cutting sites. PCR products containing CpG dinucleotides and at least 1 *Bst*UI restriction site were digested with *Bst*UI (New England BioLabs) that recognizes the sequence 5′-CGCG-3′, retained in the bisulfite-treated methylated DNA, but not in the unmethylated DNA. The DNA digests were separated by denaturing high-performance liquid chromatography (dHPLC) (Wave 1100, Transgenomic, Omaha, NE).

In case of methylated CpG dinucleotides, after enzymatic digestion, the 235 bp *PCDHAC2* PCR product, encompassing the promoter region − 43 to + 192 bp from the transcription start site, provides two fragments of 210 and 25 bp, respectively; the 290 bp *PCDHGC5* PCR product, encompassing the promoter region − 3287 to − 2997 bp upstream from the transcription start site, provides two fragments of 200 and 90 bp, respectively; the 196 bp *PCDH10* PCR product, encompassing the promoter region − 1204 to − 1008 bp upstream from the transcription start site, has two cutting sites for *Bst*UI and provides three fragments of 113, 52 and 31 base pairs, respectively. For *PCDH10*, the presence of two cutting sites for *Bst*UI restriction enzyme hampered the interpretation of the analysis in case of partial methylation of the analyzed CpG islands. For this reason, we used BGS for this gene in all cases analyzed. Also for *PCDHAC2* and *PCDHGC5* DNA from a representative tumor and non-neoplastic sample were subjected to bisulfite genomic sequencing (BGS) to verify COBRA results independently.

#### BGS

We directly sequenced the PCR products generated from bisulfite-treated templates with the same primers used for amplification (Table 2).

Sequencing analysis was performed using an ABI PRISM 3100 Genetic Analyzer (Applied Biosystems). Methylation status was expressed as the percentage of CpG methylated over the total number of CpG included in the sequence analyzed. In some cases, sequence analysis of bisulphite-treated DNA showed the simultaneous presence of both peaks (T and C), but in these cases there was always a major peak accounting for at least 70% of the total signal. This major peak was considered to call the island as methylated (C major peak) or unmethylated (T major peak) in subsequent analyses. For *PCDH10*, in cases showing CpG dinucleotides with the simultaneous presence of both peaks (T and C), we also analyzed data taking into account the relative height of the two peaks. The inclusion of this information in the analyses introduced marginal variations (2–3%) in the percentage of methylation status, and the subsequent analyses of the association between methylation and prognosis yielded virtually identical results.

### Analysis of *PCDH10* expression by RT-PCR in cancer cell lines

Total RNA was extracted from Capan-2, AsPC-1, AGS, MB-231 cancer cell lines using Trizol reagent (Invitrogen Corp., Carlsbad, California, USA. Complementary DNA (cDNA) was synthesised as previously described [19] and amplified for *PCDH10* gene with previously published primers [20]. *PCDH10* cDNA RT-PCR amplified fragments were separated by dHPLC.

#### Statistical analysis

A cut-off of 52% was chosen to dichotomize *PCDH10* methylation levels according to the receiver operating characteristic (ROC) curve analysis. Consequently, the tumor was identified as *PCDH10*^High^ with methylation levels above the cut-off threshold and *PCDH10*^Low^ with methylation levels below the threshold. The relationships between *PCDH10* methylation status and clinicopathological parameters were investigated by Pearson’s χ^2^ test.

Progression-free survival (PFS) was defined as the time from surgery to relapse, and overall survival (OS) as the time until death from any cause. Survival curves were plotted by the Kaplan-Meier method (log-rank test). Univariate analysis of *PCDH10* methylation status with outcome was tested by Cox’s proportional hazards model. SPSS Version 15.0 (SPSS, Chicago, IL) was used for statistical analyses.

## Results

### Querying public database for genes most frequently mutated in PDAC

To select genes that may play a key role in PDAC, we analyzed PDAC data in The Cancer Genome Atlas (TCGA) provisional database (accessed January 17, 2014) to identify functionally related gene groups frequently mutated in this tumor. Protocadherins were among the most frequently mutated genes in PDAC samples analyzed by TCGA (Table 3). We then used DAVID bioinformatics resources (http://david.abcc.ncifcrf.gov) to identify enriched biological themes and functional-related gene groups among the top 43 genes with > 10 mutations in PDAC according to TCGA. This analysis indicated that “Cadherins” including *PCDH10*, *PCDHGC5*, *PCDH15*, *PCDHAC2*, *CDH10* were among functional-related gene groups statistically enriched after Bonferroni (*P* = 0.014) and Benjamini (*P* = 0.004) corrections (Table 4). Notably, the enrichment of the term “Cadherins” was confirmed in a more recent analysis (April 18, 2018) in which we included the top 424 genes with > 10 mutations in PDAC, merging mutational data from different databases, including TCGA, International Cancer Genome Consortium (ICGC), Queensland Centre for Medical Genomics (QCMG), UTSouthwestern Medical Center (UTSW) (UP_SEQ_FEATURE domain: Cadherin 5; Bonferroni *P* = 1.8 10^− 8^; Benjamini *P* = 8.1 10^− 10^). Based on the above results, in this study we analyzed CpG methylation for three genes that appeared to be frequently mutated in PDAC, including *PCDHGC5* and *PCDHAC2* that had not been studied before for epigenetic modifications and *PCDH10,* whose promoter methylation had been previously suggested as a prognostic marker in other cancers [13].

**Table 3:** Top genes with > 10 mutations in PDAC tumors from TCGA (provisional, accessed January 17, 2014)

Gene	Cytoband	Gene size (Nucleotides)	n. Mutations
TP53	17p13.1	3924	37
PCDHGC5	5q31	4641	36
KRAS	12p12.1	7302	33
ZFHX3	16q22.3	17,503	26
PCDHAC2	5q31	5970	26
TCF20	22q13.3|22q13.3	7548	24
CHD3	17p13.1	9758	16
PCDH15	10q21.1	14,967	16
GIGYF1	7q22	6709	15
PCDH10	4q28.3	5516	15
ANK3	10q21	22,521	14
MED15	22q11.2	10,374	14
GZF1	20p11.21	5495	14
KIAA0907	1q22	5635	14
TTBK2	15q15.2	11,642	13
SUPT6H	17q11.2	10,615	13
GRM1	6q24	7272	13
CDKN2A	9p21	4400	13
RANGAP1	22q13	5463	13
CHD4	12p13	7474	12
ZFC3H1	12q21.1	9670	12
TAOK2	16p11.2	9058	12
SIPA1L1	14q24.2	12,412	12
HOXA1	7p15.3	2539	12
PASD1	Xq28	4429	12
NOS1AP	1q23.3	8449	12
ZMIZ1	10q22.3	10,735	12
TMCC1	3q22.1	7912	12
MAMLD1	Xq28	5958	12
SMARCC2	12q13.2	8555	11
CDH10	5p14.2	3660	11
FTSJ3	17q23.3	5213	11
MUC4	3q29	21,325	11
MED12L	3q25.1	12,619	11
TCHH	1q21.3	6900	11
MAGEC1	Xq26	4270	11
NAV2	11p15.1	14,577	11
RSPH6A	19q13.3	2547	11
FUZ	19q13.33	2951	11
SF3A1	22q12.2	6327	11
CDC27	17q21.32	6823	11
FOXN3	14q31.3	11,033	11
CXXC1	18q12	4319	11

**Table 4:**
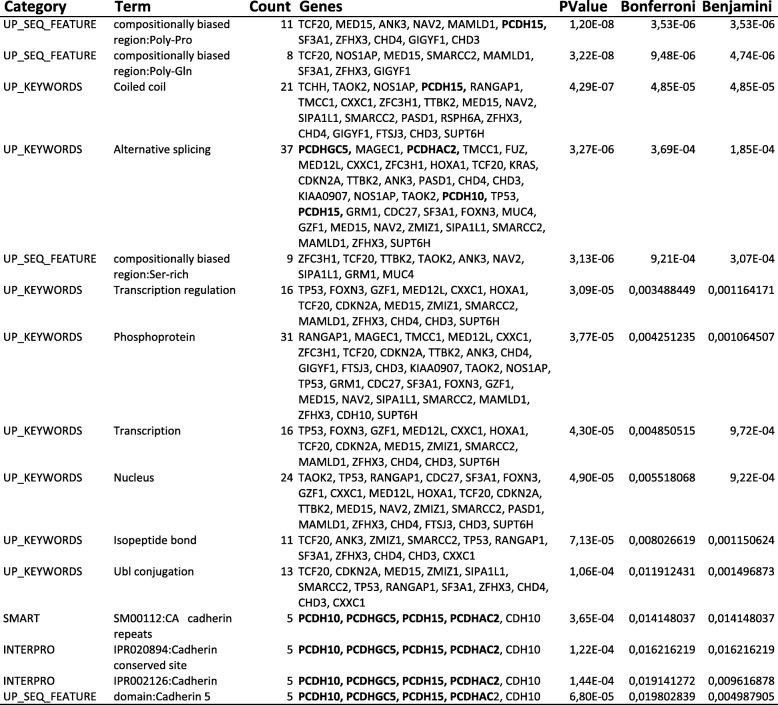
Most significant enriched biological themes and functional-related gene groups identified by DAVID in PDAC tumors from TCGA

List of most significant enriched biological themes and functional-related gene groups identified by DAVID (http://david.abcc.ncifcrf.gov) among the top 43 mutated genes with > 10 mutations in 66 PDAC tumors from The Cancer Genome Atlas database (TGCA, provisional, accessed January 17, 2014). The top terms included “SM00112:CA cadherin repeats (SMART)”, “IPR0020894:Cadherin conserved site” (INTERPRO)*,* “IPR002126:Cadherin” (INTERPRO), “domain:Cadherin 5” (UP SEQ FEATURE)*.* The table is ordered according to Bonferroni correction and all included terms were statistically significant after Bonferroni and Benjamini corrections. Protocadherin genes recurring in the table are in bold

### Methylation analysis of *PCDHAC2*, *PCDHGC5* and *PCDH10*

An exploratory study of methylation analysis on *PCDHAC2*, *PCDHGC5* and *PCDH10* was carried out in 11 pancreatic adenocarcinomas.

**Fig. 1:**
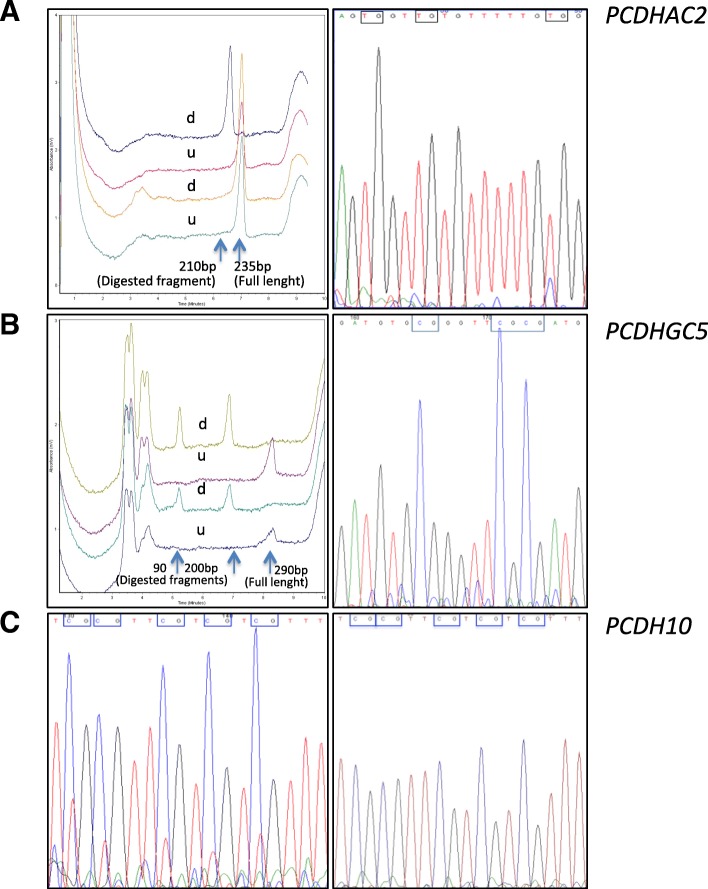
Examples of methylation analysis of PDAC cases. Panels **a** and **b** include COBRA (left) and BGS (right) analyses. In COBRA analyses, the upper two chromatograms show the digested (d) and undigested (u) methylated control, while the bottom two chromatograms (d and u) correspond to a representative case. Panel C shows BGS analyses of a representative case and a *PCDH10*-methylated cell line. **a** *PCDHAC2*. COBRA analysis shows only the full-length fragment (235 bp) in the digested sample (d) indicating the absence of CpG methylation in the case (bottom), while the corresponding methylated control (d) showed only a 210 bp fragment indicating complete digestion (top). BGS analysis confirms a substantial lack of CpG methylation, albeit one CpG dinucleotide shows a minor C peak indicating modest methylation that was also observed in non-neoplastic tissue. **b** *PCDHGC5*. COBRA analysis reveals CpG methylation as indicated by the complete cut the full-length fragment (290 bp) in two fragments (200 and 90 bp) of the digested (d) samples deriving from a representative case (bottom) and the methylated control (top). BGS analysis confirms that CpG dinucleotides are methylated. **c** *PCDH10. Left*. BGS analysis in a representative case shows major C peaks together with minor T peaks in CpG dinucleotides indicating preponderant methylation of the island. *Right*. BGS analysis of Capan-2 cell line shows that all CpG dinucleotides are fully methylated

For *PCDHAC2*, dHPLC analysis of COBRA showed in all cases only the presence of the full-length fragment in tumors, indicating that the fragment was not cut because the C in the cutting site was unmethylated and thus converted to T by bisulfite treatment abolishing the cutting site (Fig. 1a, left panel). The same pattern indicating the absence of CpG methylation was observed in the non-neoplastic pancreatic tissues analyzed. BGS showed the presence of a minor C peak, indicating modest methylation, in three of nine CpG dinucleotides sequenced both in tumor and non-neoplastic tissue (Fig. 1a, right panel). These results confirmed the monomorphic pattern of methylation in tumor and non-neoplastic tissues and the lack of relevant CpG methylation indicated by COBRA. As far as *PCDHGC5*, analysis by COBRA indicated that CpG dinucleotides were methylated in the amplicon analyzed both in tumor (Fig. 1b, left panel) and non-neoplastic tissues. BGS agreed with this finding (Fig. 1b, right panel). For *PCDH10,* in the exploratory BGS analysis, the pattern of methylation in tumors among cases was different, with six cases showing lack of methylation and five cases showing > 50% methylation of CpG dinucleotides, whereas non-neoplastic pancreatic tissues resulted unmethylated. Overall, in the exploratory analysis, both for *PCDHAC2* and *PCDHGC5* all cases had similar patterns of methylation (Table 5) providing no indicator that could be related to clinicopathological features. Conversely, for *PCDH10* the 11 cases analyzed in the exploratory study had different patterns of methylation, providing an indicator that could be related to clinicopathological features. Therefore, we extended BGS analysis of *PCDH10* to the whole series of 23 pancreatic adenocarcinomas available (Table 5). This extended analysis revealed that tumors derived from nine cases resulted not methylated and 14 methylated, with a percentage of methylation ranging from 8 to 91%, and with a mean ± SE of 55.0 ± 7.8 (Fig. 1c, left panel, Tables 5 and 6). In all non-neoplastic pancreatic tissues analyzed *PCDH10* resulted unmethylated. Sequencing of the human pancreatic carcinoma cell line Capan-2 showed complete methylation of *PCDH10* CpG dinucleotides, as expected for this control cell line (Fig. 1c, right panel).

**Table 5:** *PCDHAC2, PCDHGC5* and *PCDH10* methylation status according to clinicopathological features of patients (*n* = 23)

Sample ID	Age range (yr)	Tumor stage	Methylation status (%)		
			PCDHAC2	PCDHGC5	PCDH10
PKCH2207 T	65–70		0	100	0
PKCH2007 T	60–65	II	0	100	0
PKCH2807 T	55–60	III	0	100	0
PKCH2908 T	75–80	III	0	100	0
PKCH3708 T	45–50	III	0	100	0
PKCH3808 T	75–80	III	0	100	0
PKCH12411 T	55–60		0	100	50
PKCH13311 T	65–70	III	0	100	50
PKCH2607 T	75–80	II	0	100	50
PKCH2507 T	70–75	III	0	100	50
PKCH3408 T	75–80	III	0	100	55
PKCH21913 T	60–65	III			0
PKCH5309 T	50–55	II			0
PKCH20212 T	55–60	III			0
PKCH17112 T	70–75	III			8
PKCH14511 T	50–55	III			9
PKCH20712 T	70–75	II			9
PKCH17612 T	55–60	III			65
PKCH14111 T	65–70	II			80
PKCH10410 T	70–75	III			83
PKCH8510 T	45–50	III			80
PKCH9610 T	70–75	III			90
PKCH15511 T	35–40	II			91

**Table 6:** *PCDH10* methylation status according to clinicopathological features of patients (*n* = 23)

Variable	*PCDH10*		
	Low:	High:	*P*°
	n (%)	n (%)	
Gender			
Male	7 (43.8)	4 (57.1)	0.554
Female	9 (56.3)	3 (42.9)	
Jaundice			
No	4 (25.0)	5 (71.4)	0.036^*^
Yes	12 (75.0)	2 (28.6)	
Vascular invasion			
No	15 (93.8)	7 (100.0)	0.499
Yes	1 (6.3)	0 (0.0)	
Neural invasion			
No	13 (81.3)	5 (71.4)	0.621
Yes	3 (18.8)	2 (28.6)	
LN metastasis			
No	4 (25.0)	2 (28.6)	1.000
Yes	12 (75.0)	5 (71.4)	
Stage			
II	4 (28.6)	2 (28.6)	1.000
III	10 (71.4)	5 (71.4)	

^°^Pearson’s χ2 test

^*^Statistically significant

**Fig. 2:**
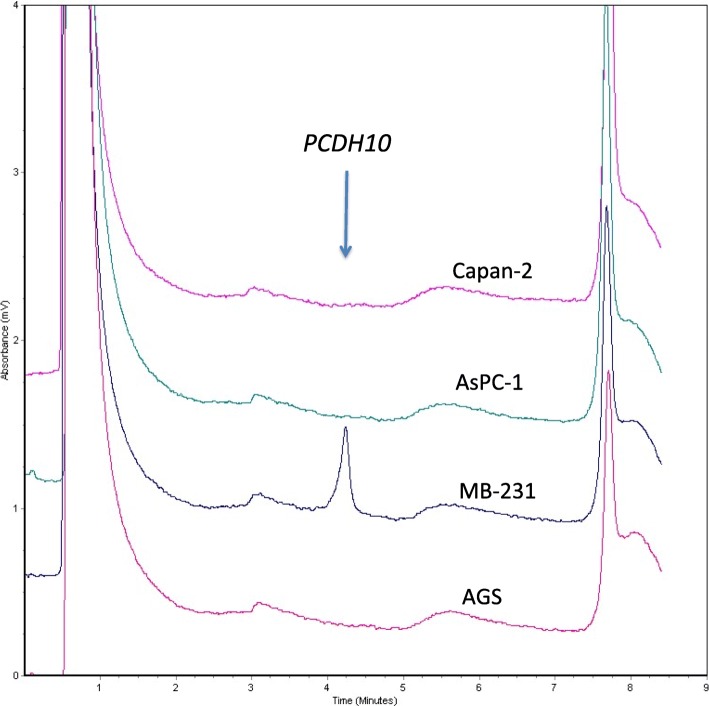
RT-PCR analysis of *PCDH10* in human cancer cell lines. dHPLC analysis of RT-PCR amplified cDNA shows a peak indicating the expression of *PCDH10* in the breast adenocarcinoma MB-231 cells, in line with the lack of methylation of the corresponding promoter in the same cells. Conversely, no peaks are detected in Capan-2 and AsPC-1 pancreatic adenocarcinoma and in AGS gastric adenocarcinoma cells, indicating lack of *PCDH10* mRNA expression in these cell lines, whose *PCDH10* promoter is fully methylated

Since RNA samples from tissues of the patients analyzed were not available, we analyzed cDNA from pancreatic (Capan-2, AsPC-1) and gastric (AGS) cancer cell lines fully methylated for *PCDH10*, as well from a breast cancer cell line (MB-231) unmethylated for *PCDH10*, to assess whether methylation status of *PCDH10* CpG dinucleotides was associated with effects on the expression of the corresponding transcript. In line with methylation status, Capan-2, AsPC-1 and AGS cell lines fully methylated for *PCDH10* did not express the corresponding mRNA, whereas the cell line MB-231 unmethylated for *PCDH10* expressed the corresponding transcript (Fig. 2).

### Hypermethylation of *PCDH10* correlates with poor prognosis in PDAC patients

**Fig. 3:**
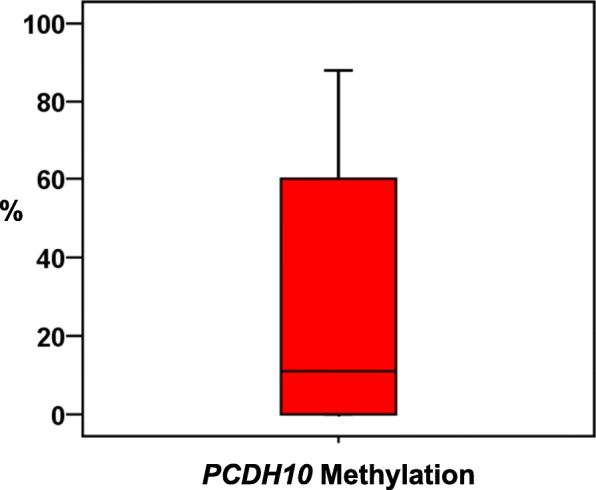
Box-and-whisker diagrams of the percentage of *PCDH10* methylation. Upper and lower ends of boxes represent 75th and 25th percentiles. The median value is showed with a solid line

Fourteen out of 23 (60.9%) tumors showed *PCDH10* methylation. In these cases, the percentages of methylation ranged from 8 to 91%, with a mean ± SE of 55.0 ± 7.8. The box-and-whisker diagram shows the *PCDH10* methylation levels registered among 23 PDAC cases (Fig. 3).

By ROC curve analysis, cases were dichotomized according to *PCDH10* methylation status: tumors with methylation levels above 52% (*n* = 7) were considered *PCDH10*^*High*^, and those with methylation levels below the cut-off value were considered *PCDH10*^*Low*^ (*n* = 16). By chi-square test, *PCDH10* methylation status was found inversely correlated with the clinical presentation of jaundice (*P* = 0.036) (Table 7).

**Table 7:** Risk of progression and death associated with the *PCDH10* methylation status

Outcome	*PCDH10* methylation status		
	HR^a^	95% CI	*P*
PFS	4.0	1.3–12.3	0.016
OS	1.8	0.6–4.9	0.263

^a^Hazard Ratio of high versus low levels of *PCDH10* methylation

**Fig. 4:**
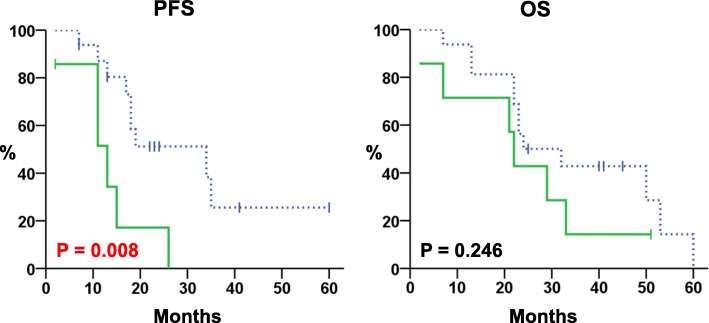
Kaplan-Meier estimates (PFS and OS) in PDAC patients (*n* = 23). Green solid lines and blue dashed lines indicate cases with high (*n* = 7) and low (*n* = 16) methylation levels of *PCDH10*, respectively

A disease progression was observed in 85.7% (6/7) of patients with *PCDH10*^High^ and 56.3% (9/16) of those with *PCDH10*^Low^ tumors. Death rates were 85.7 and 75.0% for patients with high and low methylation of *PCDH10*, respectively. At Kaplan–Meier analysis, *PCDH10*^High^ was significantly associated with worse PFS rates (*P* = 0.008), but not with OS (Fig. 4).

Univariate analysis revealed that *PCDH10*^High^ was a prognostic factor influencing PFS (HR = 4.0: 95% CI, 1.3–12.3; *P* = 0.016), but not the OS (Table 7).

## Discussion

PDAC is one of the worst malignant tumors, which commonly has an unfavourable prognosis. Currently, the most important clinical prognostic indicators of disease outcome are the PDAC staging based on the size and extent of the primary tumor and presence and extent of metastasis. Beyond the parameters used in the stage grouping (i.e., TNM classification), no additional prognostic factors are recommended for clinical care of PDAC patients. Thus, additional prognostic biomarkers are needed to provide a better risk assessment.

Recent studies showed that PDAC is not a clinically homogeneous disease, but molecularly defined subsets of patients with distinct clinical features, including prognosis and response to therapy, can be identified by integrated genome-wide analyses [4–6]. Among the four major PDAC driver genes (*KRAS*, *CDKN2A*, *TP53*, *SMAD4*), mutations in *SMAD4* were associated with poor survival, whereas mutations in *KRAS*, *CDKN2A* and *TP53*, or the presence of multiple (> 4) mutations or homozygous deletions among the most frequently mutated genes were not associated with survival [21].

In addition to mutations, epigenetic modifications may play an important role in PDAC as suggested by the observation that aberrant CpG island methylation of *reprimo*, a gene involved in p53-induced G2 cell cycle arrest, was shown to associate with worse prognosis [22]. However, the characterization of epigenetic changes occurring in PDAC has not been extensively studied, and the only genome-wide study of promoter methylation in PDAC analyzed primarily cell lines [7].

Since genes that are frequently mutated in PDAC may be crucial for the biology of this tumor, and they might also be a target of epigenetic dysregulation, we searched for genes frequently mutated in PDAC by querying The Cancer Genome Atlas (TCGA) provisional database. The bioinformatics analysis identified protocadherins among the most mutated genes in PDAC.

Therefore, we evaluated whether the epigenetic differences in terms of promoter methylation of protocadherins between the tumor and non-tumor tissue samples could be used as survival predictors in PDAC patients. In particular, we studied the promoter methylation of *PCDHAC2, PCDHGC5* and *PCDH10* because they emerged among the most mutated genes in PDAC through the aforementioned unbiased in silico approach. Notably, the methylation status of *PCDHAC2* and *PCDHGC5* were never analyzed before in PDAC, while *PCDH10* had been previously studied in PDAC cancer cell lines [7] and one study analyzed this gene in PDAC primary tumors [14].

In our study *PCDHAC2* resulted hypomethylated, whereas *PCDHGC5* was hypermethylated in all PDAC samples and the same patterns of methylation were also observed in matched adjacent non-neoplastic pancreatic tissues, suggesting that CpG promoter methylation of these genes does not play a major role in the biology of this tumor. Conversely, *PCDH10*, that resulted unmethylated in adjacent non-neoplastic pancreatic tissues showed a variable degree of methylation ranging from high to low levels in matched PDAC samples. As expected, *PCDH10* methylation status correlated with the lack of expression of the corresponding transcript in *PCDH10* fully methylated cancer cell lines and, conversely, with expression of *PCDH10* in the unmethylated cell line analyzed. In line with our findings, a previous study [14] found a significant correlation between *PCDH10* methylation and loss of *PCDH10* mRNA expression in pancreatic, gastric and colorectal cancers tissues.

The variability of *PCDH10* methylation among patients led us to investigate the possible correlations between CpG dinucleotide methylation in this gene and PDAC clinical outcome. In this analysis we found, for the first time, an association between *PCDH10* promoter methylation status and PDAC patients outcomes, being the hypermethylation of the gene associated with shorter progression-free survival.

Deaths occurred at high rates in both cohorts of PDAC patients and the percentage tended to be higher among PDAC patients with *PCDH10*^High^ rather than *PCDH10*^Low^ tumors (86% versus 75%, respectively). However, possibly because of the high rates of death, the relatively small differences among cohorts and the limited number of patients analyzed, we did not find any correlation between *PCDH10* status and overall survival.

*PCDH10* was already reported to be inactivated by promoter methylation in various types of cancer, including non-small cell lung cancer [23], gastric cancer [24], colorectal cancer [25], nasopharyngeal, esophageal [17], endometrioid endometrial carcinoma [26, 27], bladder cancer [28], cervical cancer [29], suggesting that it plays an oncosuppressor role in those tumors. In support of a role for *PCDH10* as an oncosuppressor gene, re-expression of this gene by transfection in a gastric cancer cell line inhibited the proliferation, migration, invasion ability, as well as its tumor growth in mice [16]. Further evidence that this gene plays an oncosuppressor role derives from the observation that methylation of *PCDH10* was associated with poor prognosis in patients with gastric cancer [16]. In line with this evidence, the genetic deletion of *PCDH10* represents an adverse prognostic marker for the survival of patients with CRC [30]. In pancreatic tumors, however, the potential role of *PCDH10* as oncosuppressor gene in PDAC was investigated only in pancreatic cancer cell lines where this gene was silenced by methylation and its re-expression by transfection inhibited the proliferation, migration, invasion ability and induced apoptosis [31]. The only study which analyzed *PCDH10* methylation in pancreatic tumor samples failed to find any correlation between *PCDH10* methylation status and PDAC staging, which was the pathologic feature analyzed in that study [14]. Also in our study there was no correlation between methylation and tumor staging, but we found that this epigenetic modification was correlated with PFS, which had not been previously analyzed.

## Conclusions

Promoter methylation has been reported as a promising predictive biomarker in many human cancers. However, a better understanding of the specific epigenetic changes affecting the prognosis of PDAC is necessary.

In our study, we identified for the first time that methylation status of *PCDH10* can predict the patients’ prognosis and may have a significant impact on the outcome in terms of progression-free survival of the patients with PDAC. In particular, high levels of *PCDH10* promoter methylation could be useful to identify patients at high risk of disease progression and early death after surgical treatment, contributing to a more accurate stratification of PDAC patients for personalized clinical management.

## Abbreviations

BGS: Bisulfite genomic sequencing
COBRA: Combined Bisulfite Restriction Analysis
dHPLC: denaturing high-performance liquid chromatography
OS: Overall survival
PDAC: Pancreatic ductal adenocarcinoma
PFS: Progression-free survival
ROC: Receiver operating characteristic

## References

[1] Bray F, Ferlay J, Soerjomataram I, Siegel RL, Torre LA, Jemal A (2018). Global cancer statistics 2018: GLOBOCAN estimates of incidence and mortality worldwide for 36 cancers in 185 countries. CA Cancer J Clin.

[2] Cid-Arregui A, Juare V (2015). Perspectives in the treatment of pancreatic adenocarcinoma. World J Gastroenterol.

[3] Waddell N, Pajic M, Patch AM, Chang DK, Kassahn KS, Bailey P (2015). Whole genomes redefine the mutational landscape of pancreatic cancer. Nature..

[4] Witkiewicz AK, McMillan EA, Balaji U, Baek G, Lin WC, Mansour J (2015). Whole-exome sequencing of pancreatic cancer defines genetic diversity and therapeutic targets. Nat Commun.

[5] Bailey P, Chang DK, Nones K, Johns AL, Patch AM, Gingras MC (2016). Genomic analyses identify molecular subtypes of pancreatic cancer. Nature..

[6] Biankin AV, Waddell N, Kassahn KS, Gingras MC, Muthuswamy LB, Johns AL (2012). Pancreatic cancer genomes reveal aberrations in axon guidance pathway genes. Nature.

[7] Vincent A, Omura N, Hong SM, Jaffe A, Eshleman J, Goggins M (2011). Genome-wide analysis of promoter methylation associated with gene expression profile in pancreatic adenocarcinoma. Clin Cancer Res.

[8] Jones S, Zhang X, Parsons DW, Lin JC, Leary RJ, Angenendt P (2008). Core signaling pathways in human pancreatic cancers revealed by global genomic analyses. Science..

[9] Wu Q, Zhang T, Cheng JF, Kim Y, Grimwood J, Schmutz J (2001). Comparative DNA sequence analysis of mouse and human protocadherin gene clusters. Genome Res.

[10] Frank M, Kemler R (2002). Protocadherins. Curr Opin Cell Biol.

[11] Morishita H, Yagi T (2007). Protocadherin family: diversity, structure, and function. Curr Opin Cell Biol.

[12] Men Mah K, Weiner JA (2017). Regulation of Wnt signaling by protocadherins. Semin Cell Dev Biol.

[13] El Hajj N, Dittrich M, Haaf T (2017). Epigenetic dysregulation of protocadherins in human disease. Semin Cell Dev Biol.

[14] Yu B, Yang H, Zhang C, Wu Q, Shao Y, Zhang J, Guan M, Wan J, Zhang W (2010). High-resolution melting analysis of PCDH10 methylation levels in gastric, colorectal and pancreatic cancers. Neoplasma..

[15] Hamilton SR, Aaltonen LA (2000). World Health Organization classification of Tumours. Pathology and genetics of Tumours of the digestive system.

[16] Yu J, Cheng YY, Tao Q, Cheung KF, Lam CN, Geng H, Tian LW, Wong YP, Tong JH, Ying JM, Jin H, Chan FK, Sung JJ, To KF (2009). Methylation of protocadherin 10, a novel tumor suppressor, is associated with poor prognosis in patients with gastric cancer. Gastroenterology..

[17] Ying J, Li H, Seng TJ, Langford C, Srivastava G, Tsao SW, Putti T, Murray P, Chan AT, Tao Q (2006). Functional epigenetics identifies a protocadherin PCDH10 as a candidate tumor suppressor for nasopharyngeal, esophageal and multiple other carcinomas with frequent methylation. Oncogene..

[18] Li LC, Dahiya R (2002). MethPrimer: designing primers for methylation PCRs. Bioinformatics..

[19] Aceto GM, Fantini F, De Iure S, Di Nicola M, Palka G, Valanzano R, Di Gregorio P, Stigliano V, Genuardi M, Battista P, Cama A, Curia MC (2015). Correlation between mutations and mRNA expression of APC and MUTYH genes: new insight into hereditary colorectal polyposis predisposition. J Exp Clin Cancer Res.

[20] Xu Y, Yang Z, Yuan H, Li Z, Li Y, Liu Q, Chen J (2015). PCDH10 inhibits cell proliferation of multiple myeloma via the negative regulation of the Wnt/β-catenin/BCL-9 signaling pathway. Oncol Rep.

[21] Blackford A, Serrano K, Wolfgang CL, Parmigiani G, Jones S, Zhang X (2009). SMAD4 gene mutations are associated with poor prognosis in pancreatic Cancer. Clin Cancer Res.

[22] Sato N, Fukushima N, Matsubayashi H, Iacobuzio-Donahue CA, Yeo CJ, Goggins M (2006). Aberrant methylation of Reprimo correlates with genetic instability and predicts poor prognosis in pancreatic ductal adenocarcinoma. Cancer..

[23] Tang X, Yin X, Xiang T, Li H, Li F, Chen L, Ren G (2013). Protocadherin 10 is frequently downregulated by promoter methylation and functions as a tumor suppressor gene in non-small cell lung cancer. Cancer Biomark.

[24] Li Z, Chim JC, Yang M, Ye J, Wong BC, Qiao L (2012). Role of PCDH10 and its hypermethylation in human gastric cancer. Biochim Biophys Acta.

[25] Zhong X, Shen H, Mao J, Zhang J, Han W (2017). Epigenetic silencing of protocadherin 10 in colorectal cancer. Oncol Lett.

[26] Zhao Y, Yang Y, Trovik J, Sun K, Zhou L, Jiang P, Lau TS, Hoivik EA, Salvesen HB, Sun H, Wang H (2014). A novel wnt regulatory axis in endometrioid endometrial cancer. Cancer Res.

[27] Yang Y, Jiang Y, Jiang M, Zhang J, Yang B, She Y, Wang W, Deng Y, Ye Y (2016). Protocadherin 10 inhibits cell proliferation and induces apoptosis via regulation of DEP domain containing 1 in endometrial endometrioid carcinoma. Exp Mol Pathol.

[28] Lin YL, Li ZG, He ZK, Guan TY, Ma JG (2012). Clinical and prognostic significance of protocadherin-10 (PCDH10) promoter methylation in bladder cancer. J Int Med Res.

[29] Narayan G, Scotto L, Neelakantan V, Kottoor SH, Wong AH, Loke SL, Mansukhani M, Pothuri B, Wright JD, Kaufmann AM, Schneider A, Arias-Pulido H, Tao Q, Murty VV (2009). Protocadherin PCDH10, involved in tumor progression, is a frequent and early target of promoter hypermethylation in cervical cancer. Genes Chromosomes Cancer.

[30] Tzu-Ming J, Ming-Hong T, Hoi-Yan L, Wei-Ting W, Chun-Chieh C, Sheng-Tai T (2014). Protocadherin 10 suppresses tumorigenesis and metastasis in colorectal cancer and its genetic loss predicts adverse prognosis. Int J Cancer.

[31] Qiu C, Bu X, Jiang Z (2016). Protocadherin-10 acts as a tumor suppressor gene, and is frequently downregulated by promoter methylation in pancreatic cancer cells. Oncol Rep.

